# A potential bat adenovirus-based oncolytic virus targeting canine cancers

**DOI:** 10.1038/s41598-021-96101-4

**Published:** 2021-08-18

**Authors:** Hiromichi Matsugo, Tomoya Kitamura-Kobayashi, Haruhiko Kamiki, Hiroho Ishida, Wataru Sekine, Akiko Takenaka-Uema, Takayuki Nakagawa, Shin Murakami, Taisuke Horimoto

**Affiliations:** 1 Laboratory of Veterinary Microbiology, Graduate School of Agricultural and Life Sciences, University of Tokyo, Tokyo, Japan; 2 Laboratory of Veterinary Surgery, Graduate School of Agricultural and Life Sciences, University of Tokyo, Tokyo, Japan

**Keywords:** Virology, Cancer therapy

## Abstract

Although a canine adenovirus (CAdV)-based oncolytic virus (OV) candidate targeting canine tumors has been reported, its oncolytic effect could be attenuated by CAdV vaccine-induced neutralizing antibodies in dog patients. To circumvent this issue, we focused on the bat adenovirus (BtAdV) strain, which was previously isolated from healthy microbats. We previously showed that this virus replicated efficiently in canine cell lines and did not serologically cross-react with CAdVs, suggesting that it may offer the possibility of an OV candidate for canine tumors. Here, we tested the growth properties and cytotoxicity of the BtAdV Mm32 strain in a panel of canine tumor cells and found that its characteristics were equivalent to those of CAdVs. To produce an Mm32 construct with enhanced tumor specificity, we established a novel reverse genetics system for BtAdV based on bacterial artificial chromosomes, and generated a recombinant virus, Mm32-E1Ap + cTERTp, by inserting a tumor-specific canine telomerase reverse transcriptase promoter into its E1A regulatory region. The growth and cytotoxicity of this recombinant were superior to those of wild-type Mm32 in canine tumor cells, unlike in normal canine cells. These data suggest that Mm32-E1Ap + cTERTp could be a promising OV for alternative canine cancer therapies.

## Introduction

Cancer is a major cause of death in domestic dogs^1–3^. Traditional treatments for cancers such as surgery, chemotherapy, and radiation therapy are sometimes insufficient to remove tumors efficiently; therefore, additional treatments are required. Oncolytic virus (OV) therapy is a promising approach that involves the application of natural or genetically modified viruses to destroy tumor cells selectively and induce anti-tumor immunity. Herpes simplex virus 1-based Talimogene laherparepvec (T-VeC) has been approved by the U.S. Food and Drug Administration (FDA) and European Medicine Agency (EMA), and human adenovirus 5 (HAdV5)-based H101 has been approved by the China Food and Drug Administration Department for clinical use in humans^4^. Furthermore, several OV candidates are currently being tested in human clinical trials. In contrast to these advances in OVs for human cancers, OVs for canine cancers have not been approved yet, and few OVs are under clinical trials^5,6^.

Adenoviruses are one of the most promising OV candidates because they can be armed with an exogenous gene to enhance its anti-tumor effects, and adenovirus stocks with high titers can be prepared in cell cultures. The adenoviral E1A and E1B proteins are necessary for efficient viral replication in normal cells, where E1A binds retinoblastoma protein (pRb), releasing transcription factor E2F, leading to efficient viral DNA replication, and E1B-55 K mediates efficient viral mRNA transport; the former function is partially complemented in tumor cells that lack pRB, and the latter function is also complemented in some tumor cells, although the molecular mechanism has not been fully understood. Therefore, deleting specific domains, such as the pRB-binding site of E1A or replacing the E1A promoter with a tumor-specific promoter, are common strategies to generate oncolytic adenoviruses^7–9^. Oncolytic adenoviruses targeting canine tumors based on HAdV5 and canine adenovirus 2 (CAdV2) have been reported previously^10–12^. Although the HAdV5-based OV could replicate in various canine tumor cells, their replication efficiencies were low, indicating limited suitability as an OV for canine cancers. In contrast, the CAdV2-based OV could replicate to high titers in canine tumor cells and is a promising OV candidate for canine cancers. However, the oncolytic CAdV2 could be attenuated by homologous neutralizing antibodies induced by widely used CAdV vaccines, similar to the weakened anti-tumor effects of oncolytic HAdV5 by homologous or cross-reactive antibodies in humans^13–15^.

To circumvent this issue, we focused on bat adenovirus (BtAdV), which belongs to the genus *Mastadenovirus*, similar to CAdVs^16^. BtAdVs have been detected worldwide, and some strains have been able to replicate in various mammalian host cells in vitro^17–21^. We have previously isolated BtAdV strains from apparently healthy microbats in Japan and found that these viruses could replicate efficiently in cell cultures of canine origin^21^, suggesting that they could replicate in canine tumor cells as well. Furthermore, they did not cross-react serologically with the CAdVs. These data suggest that our BtAdV isolates could be potential OV candidates for canine cancer therapy.

In this study, to construct a BtAdV-based OV candidate, we first established a novel reverse genetics system for BtAdVs by utilizing a bacterial artificial chromosome vector. Using this system, we generated a recombinant BtAdV mutant in which the canine telomerase reverse transcriptase promoter^22^, a tumor-specific promoter, was inserted into its E1A regulatory region, so that this recombinant virus could selectively express E1A protein and efficiently replicate in tumor cells. We then tested the replication properties and cytotoxicity of this recombinant in canine tumor cell cultures of various origins to evaluate its potential as an OV candidate for canine cancer therapy.

## Results

### Growth and cytotoxicity of BtAdV in canine tumor cell cultures

**Figure 1:**
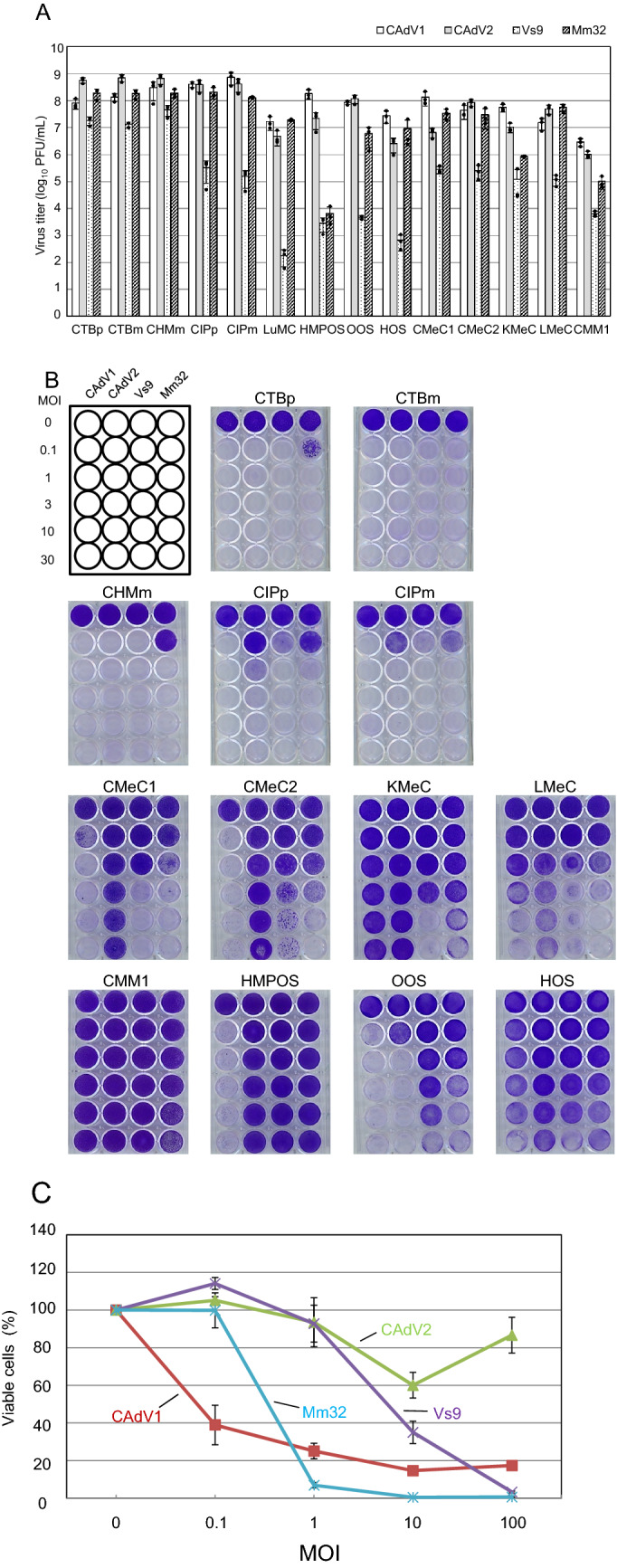
Growth and cytotoxicity of canine adenoviruses (CAdVs) and bat adenoviruses (BtAdVs) in canine tumor cells. (**A**) Canine tumor cell cultures derived from mammary tumors (CTBm, CTBp, CIPm, CIPp, and CHMm), melanomas (CMeC1, CMeC2, KMeC, LMeC, and CMM1), osteosarcomas (HMPOS, HOS, and OOS), and mast cell tumors (LuMC), were infected with CAdV1, CAdV2, Vs9, or Mm32 at a multiplicity of infection (MOI) of 5. At 3 days post-infection, the medium and cells were frozen and thawed three times, and the supernatants were collected. The viral titers were determined using plaque assay. The results are reported as the mean titers with standard errors of the mean for three independent experiments (with scatter plot of three values). Significant differences on dataset are analyzed using the Holm method. (**B**) Canine tumor cells were infected with CAdV1, CAdV2, Vs9, or Mm32 at an MOI of 0.1, 1, 3, 10, or 30. At 5 days post-infection, cells were fixed with methanol and stained with 0.1% crystal violet. (**C**) LuMC cells were infected with CAdV1, CAdV2, Vs9, or Mm32 at an MOI of 0.1, 1, 10, or 100. Cells were stained with 0.5% trypan blue, viable cells were counted, and normalized to mock-infected cells (MOI = 0). Data are presented as the mean values with standard errors of the mean for three independent experiments.

We previously showed that BtAdVs Mm32 and Vs9 strains replicated efficiently in Madin–Darby canine kidney (MDCK) cells, but did not cross-react with anti-CAdV-neutralizing antibodies^21^. To evaluate the potential of these BtAdVs as OVs in canine cancers, we examined their growth and cytotoxicity in canine tumor cells originating from mammary tumors (CTBm^23^, CTBp^23^, CIPm^23^, CIPp^23^, and CHMm^23^), melanomas (CMeC1^24^, CMeC2^24^, KMeC^24^, LMeC^24^, and CMM1^25^), osteosarcomas (HMPOS^26^, HOS^27^, and OOS^27^), and mast cell tumors (LuMC^28^). The Mm32 strain was able to replicate in all canine tumor cell cultures of mammary tumor and mast cell origins, and in most cells of other origins at levels equivalent to those of wild-type CAdV1 and CAdV2 (Fig. 1A). Compared to the Mm32 strain, the Vs9 strain replicated less efficiently, even in cells of mammary tumor and mast cell origins. In total, significantly lower growth rates (p < 0.05) of Vs9 were observed in 5 (CTBm, CIPp, OOS, KMeC, and CMM1) or 7 (CTBp, CTBm, OOS, CMeC2, KMeC, LMeC, and CMM1) of 14 canine tumor cell lines tested, compared to CAdV1 and CAdV2, respectively, whereas those of Mm32 were observed in 3 (OOS, KMeC, and CMM1) or 4 (CTBm, OOS, KMeC, and CMM1) under the same conditions. In addition, similar levels of cytotoxicity were observed with BtAdV infections when compared to that of CAdVs in most canine tumor cell cultures, whereas less cytotoxicity was observed in some cell cultures, even with wild-type CAdVs (Fig. 1B,C). These data suggest that BtAdVs, especially Mm32, may act as potential OVs for canine tumors. Therefore, we selected Mm32 to construct an OV candidate for further assessment.

### Establishment of a reverse genetics system for BtAdV

**Figure 2:**
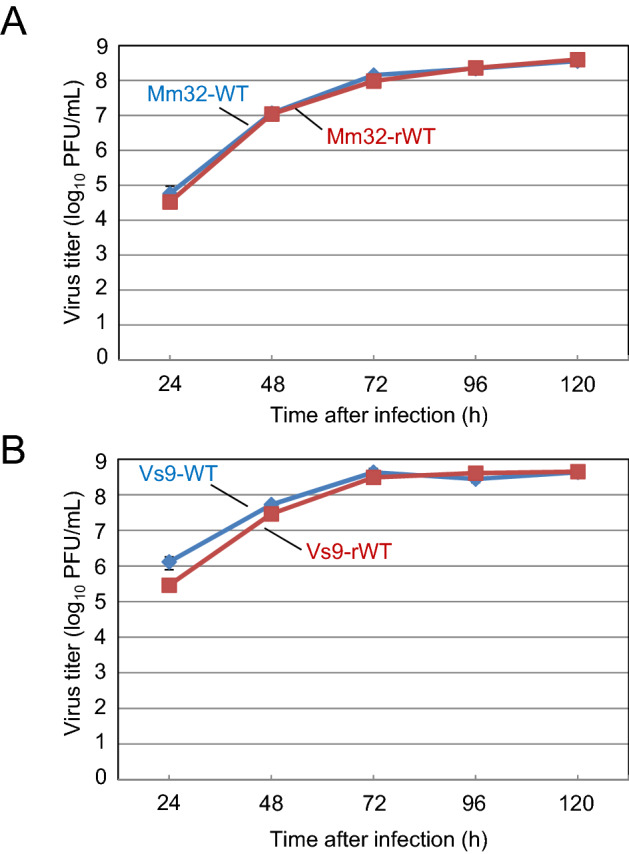
Growth kinetics of the wild-type (WT) and recombinant wild-type (rWT) viruses. MDCK cells were infected with (**A**) Mm32-WT or Mm32-rWT and (**B**) Vs9-WT or Vs9-rWT at an MOI of 0.01. The culture supernatants were collected at the indicated time points, and the viral titers were determined using plaque assays. The results are reported as the mean titers with standard errors of the mean for three independent experiments. The mixed ANOVA indicated no significance of the differences between growth kinetics of (**A**) Mm32-WT dataset vs. rWT (p = 0.814) and (**B**) Vs9-WT vs. rWT (p = 0.273).

To generate a genetically modified OV candidate, we established a novel reverse genetics system for BtAdVs using a bacterial artificial chromosome (BAC) vector. First, we constructed infectious BAC clones (pBAC-Mm32 and pBAC-Vs9) by inserting the full-length genomes of Mm32 or Vs9 into BAC vectors (Supplementary Fig. S1). The digestion profiles of these BAC clones with restriction enzymes indicated their appropriate structures without unintended constructional errors (Supplementary Fig. S2). Then, viral DNA released from pBAC-Mm32 or pBAC-Vs9 with restriction digestion was transfected into MDCK cells, resulting in cytopathic effects (CPEs) in the cells post-transfection. Restriction digestion of genomic DNA from the rescued viruses (Vs9-rWT or Mm32-rWT) in the cell supernatants showed profiles identical to those of the respective wild-type viruses (Vs9-WT or Mm32-WT) (Supplementary Fig. S2). Furthermore, the growth kinetics of Vs9-rWT and Mm32-rWT were significantly similar to those of Vs9-WT or Mm32-WT in MDCK cells (Fig. 2A,B). These data imply that BAC-based reverse genetics systems were successfully established for BtAdVs.

### Generation of E1-deleted BtAdVs

To investigate whether partial deletion or modification of the E1 region may create an OV candidate based on BtAdV, similar to other mastadenoviruses^7–9^, we generated an E1-deleted recombinant virus using Mm32 by reverse genetics. First, we constructed a manipulated pBAC-Mm32, whose E1 region was replaced with the reporter Venus gene, producing pBAC-Mm32-ΔE1-Venus (Supplementary Fig. S3). To generate E1-deleted Mm32, we prepared cells that expressed Mm32 E1 (namely MDCK-Mm32-E1-28 cells), which is required to prepare high titers of the E1-deleted virus. E1A expression in these cells was confirmed by immunofluorescence assay (Supplementary Fig. S4A), although its localization was different between plasmid-transfected and MDCK-Mm32-E1-28 cells; E1A was localized to both the nucleus and cytoplasm in the former, whereas it was predominantly localized to the nucleus in the latter. A previous report showed that E1A is mainly localized to the nucleus but also partially to the cytoplasm^29^. These distinct localization patterns of E1A may be attributed to its expression levels between these cells. Although the expression of E1B was not confirmed at the protein level due to the lack of a specific antibody, its transcript was detected by RT-PCR (Supplementary Fig. S4B). We then transfected MDCK-Mm32-E1-28 cells with an E1-deleted construct released from pBAC-Mm32-ΔE1-Venus, resulting in the generation of infectious viruses that showed CPE. Venus expression was confirmed in the rescued virus-infected cells, indicating the successful generation of E1-deleted Mm32 (namely Mm32-ΔE1-Venus) (Supplementary Fig. S3).

### Growth of E1-deleted BtAdV

**Figure 3:**
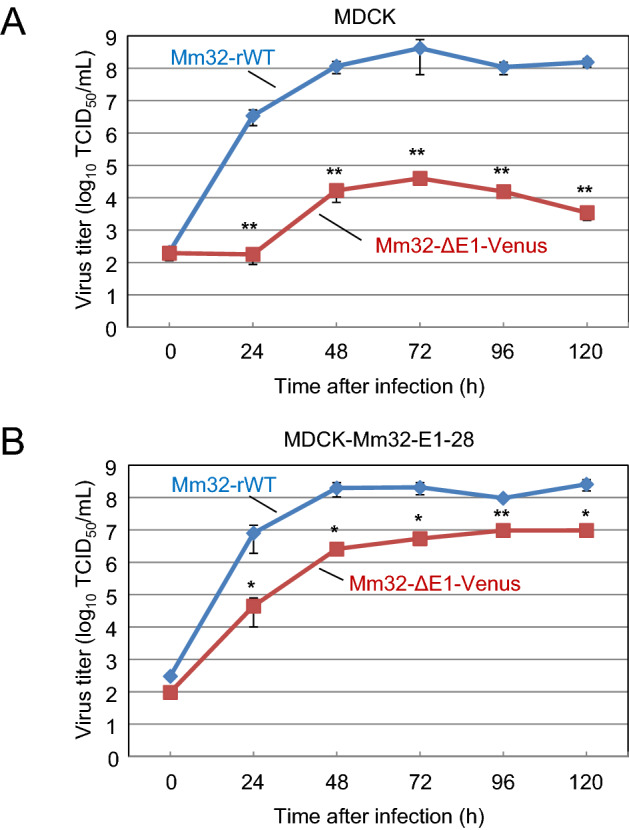
Growth kinetics of Mm32-rWT and Mm32-ΔE1-Venus. (**A**) MDCK cells and (**B**) MDCK-Mm32-E1-28 cells were infected with Mm32-rWT or Mm32-ΔE1-Venus at an MOI of 0.2, respectively. The culture supernatants were collected at the indicated time points, and the viral titers (TCID_50_/mL) were determined in MDCK-Mm32-E1-28 cells. The results are reported as the mean titers with standard errors of the mean for three independent experiments. The mixed ANOVA indicated the significance of the differences between growth kinetics of Mm32-WT vs. Mm32-ΔE1-Venus in (**A**) MDCK cells (p < 0.001) and (**B**) MDCK-Mm32-E1-28 cells (p < 0.001). The asterisks indicate significant differences at each time-point (*p < 0.05; **p < 0.01 by Holm method).

To investigate the E1-requirement of BtAdV Mm32 for efficient growth, similar to other mastadenoviruses^30,31^, the growth properties of Mm32-ΔE1-Venus were compared with those of Mm32-rWT in MDCK-WT (Fig. 3A) and MDCK-Mm32-E1-28 cells (Fig. 3B). The E1-deleted virus grew to 3–4 log lower titers than Mm32-rWT in MDCK-WT cells, whereas its growth was attenuated to less than 2 log lower titers in MDCK-Mm32-E1-28 cells, indicating that Mm32 requires E1 for efficient replication in MDCK cells. These data suggest that artificial modification of E1 expression could be applied to generate Mm32-based OV candidates with replication specificity in tumor cells.

### Construction of a tumor-enhanced promoter for E1A expression

**Figure 4:**
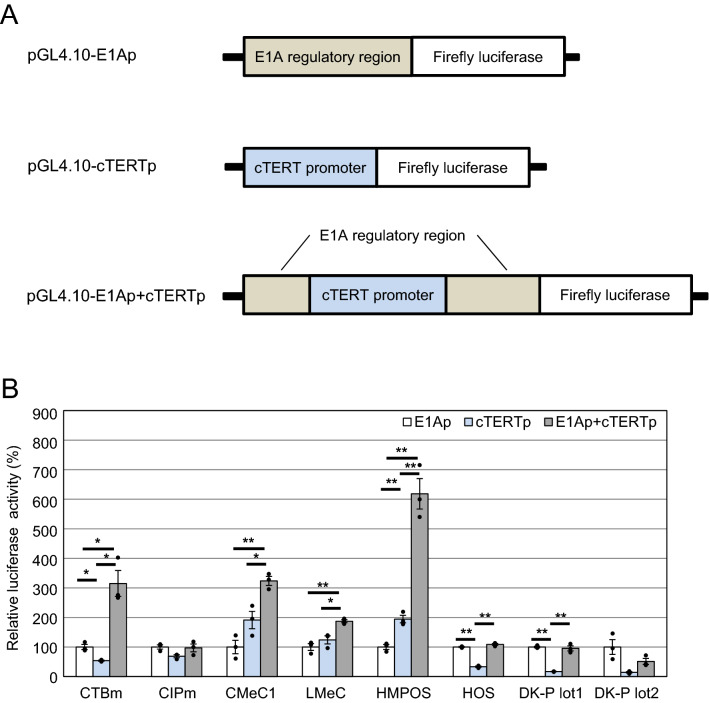
Luciferase reporter assay in various canine tumor cells and normal canine cells. (**A**) Schematic representation of the constructs. Mm32-E1A regulatory region (E1Ap), canine telomerase reverse transcriptase promoter (cTERTp), or the chimeric promoter (E1Ap + cTERTp) was cloned into pGL4.10 [luc2]. The resultant plasmids were named pGL4.10-E1Ap, pGL4.10-cTERTp, and pGL4.10-E1Ap + cTERTp, respectively. (**B**) Transcriptional activity of the E1Ap, cTERTp, and E1Ap + cTERTp. The construct with each promoter was transfected into canine tumor cells (CTBm, CIPm, CMeC1, LMeC, HMPOS, and HOS cells) and normal canine cells (DK-P lot1 and DK-P lot2 cells). At 24 h post-transfection, cell lysates were subjected to the dual-luciferase assay. Relative promoter activities are shown as the ratio of firefly luciferase to Renilla luciferase (an internal control) activity, and normalized to E1Ap activity. Data are presented as the mean values with standard errors of the mean for three independent experiments (with scatter plot of three values). The asterisks indicate significant differences (*p < 0.05; **p < 0.01 by Holm method).

To generate an Mm32-based OV candidate, we sought to replace the E1A promoter (E1Ap) with a tumor-specific promoter for E1 expression. Telomerase is a ribonucleoprotein complex composed of telomerase reverse transcriptase (TERT), telomerase RNA, and other proteins, and is responsible for telomere maintenance. A previous report showed that most canine tumor cells possessed high telomerase activity, whereas normal canine cells did not^32^. TERT expression is correlated with telomerase activity, and canine TERT promoter (cTERTp) activity is upregulated in most canine tumor cells compared to that in normal canine cells^22^. Therefore, replacement of E1Ap with cTERTp confers tumor specificity to BtAdV. However, the E1A regulatory region might contain genome packaging signals, similar to other adenoviruses^33,34^. We inserted cTERTp into the E1A regulatory region such that the packaging signal might be maintained (Fig. 4A). We tested the transcriptional activity of this chimeric promoter using a reporter assay in canine tumor cells or normal cells. As expected, cTERTp activity was drastically reduced in normal canine cells and markedly increased in tumor cells such as CMeC1 and HMPOS, compared to E1Ap activity. In contrast, the activities of the chimeric promoter were comparable to or higher than those of E1Ap in canine tumor cells (Fig. 4B). In particular, chimeric promoter activity was remarkably enhanced in CTBm, CMeC1, and HMPOS. These data suggest a synergistic effect of the chimeric construct on tumor specificity. Therefore, we selected this chimeric promoter to generate an Mm32-based OV candidate.

### Generation of BtAdV OV candidate

**Figure 5:**
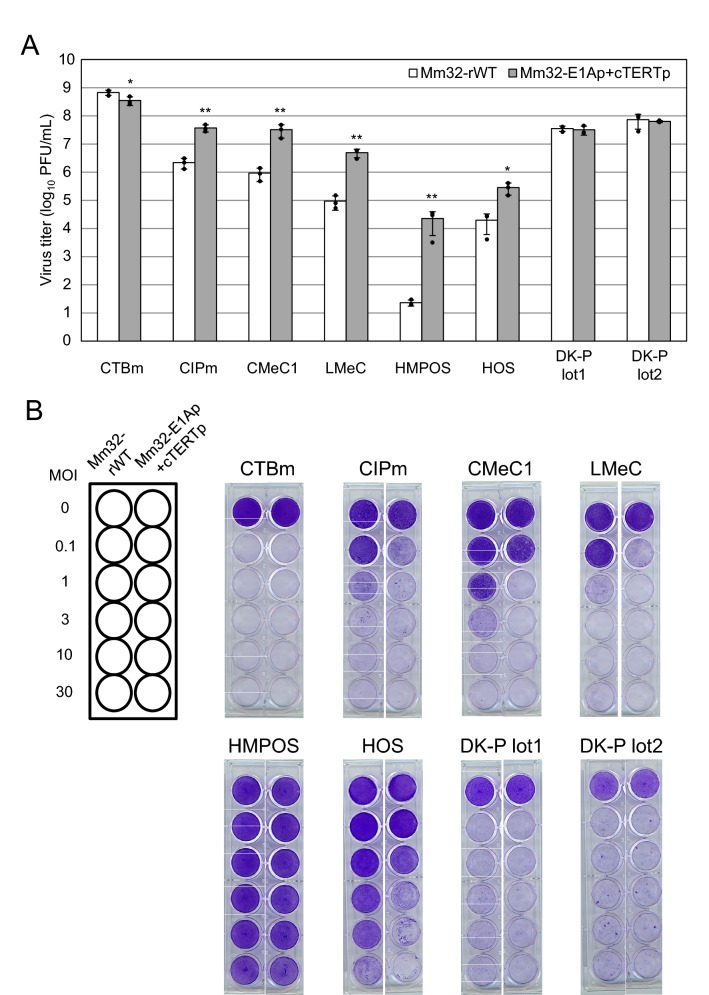
Growth and cytotoxicity of Mm32-rWT and Mm32-E1Ap + cTERTp in canine tumor cells. (**A**) Canine tumor cells (CTBm, CIPm, CMeC1, LMeC, HMPOS, and HOS) and canine kidney-derived normal cells (DK-P lot1 and DK-P lot2) were infected with Mm32-rWT or Mm32-E1Ap + cTERTp at an MOI of 0.1, respectively. At 3 days post-infection, the medium and cells were frozen and thawed three times, and the supernatants were collected. Viral titers were determined by plaque assay using MDCK-Mm32-E1-28 cells. The results are reported as the mean titers with standard errors of the mean for three independent experiments (with scatter plot of three values). The asterisks indicate significant differences (*p < 0.05; **p < 0.01 by Student’s t-test). (**B**) Canine tumor cells and normal canine cells were infected with Mm32-rWT or Mm32-E1Ap + cTERTp at an MOI of 0.1, 1, 3, 10, or 30. At 5 days post-infection, the cells were fixed with methanol and stained with 0.1% crystal violet.

We transfected MDCK-Mm32-E1-28 cells with Mm32-E1Ap + cTERTp DNA released from pBAC-Mm32-E1Ap + cTERTp, followed by generation of the infectious virus, Mm32-E1Ap + cTERTp. To investigate tumor specificity, we examined viral growth and cytotoxicity in canine tumor cells and normal cells. Mm32-E1Ap + cTERTp grew to higher titers than Mm32-rWT in most canine tumor cells, whereas it grew to similar titers to Mm32-rWT in normal canine cells (Fig. 5A). In addition, greater cytotoxicity was observed with Mm32-E1Ap + cTERTp than with Mm32-rWT in most canine tumor cells, unlike in normal canine cells (Fig. 5B). These data suggest that Mm32-E1Ap + cTERTp replicated more selectively and efficiently than wild-type Mm32 in tumor cell cultures.

## Discussion

Although the development of CAdV-based OV candidates targeting canine cancers has been previously reported^11,12^, these candidates could be attenuated by vaccine-induced anti-CAdV neutralizing antibodies in dogs that had been vaccinated with CAdV vaccines. To circumvent this issue, we focused on BtAdVs, which did not cross-react with anti-CAdVs neutralizing antibodies, and constructed a genetically modified BtAdV, in which a tumor-specific promoter was accommodated for E1A gene expression, which is essential for viral replication. To this end, we selected the BtAdV Mm32 strain, which was isolated from an apparently healthy Japanese microbat and demonstrated its efficient growth in canine tumor cell cultures, and established a BAC-based reverse genetics system to artificially generate a recombinant Mm32. Using this system, we generated a recombinant Mm32-E1Ap + cTERTp by inserting tumor-specific cTERTp into its E1A regulatory region, suggesting that this virus could be a promising OV for alternative canine tumor therapy.

In human medicine, to evade anti-HAdV neutralizing antibodies, OVs with simian adenovirus^35^ or rare HAdV serotypes^15,36^ have been reported, and the latter are now in clinical trials^37^. Similar approaches to escape from neutralizing antibodies have also been reported in other virus-based OVs^38,39^. Furthermore, as neutralizing antibodies against vectors decrease the efficiency of gene transduction, these approaches might increase the efficiency of gene transduction^40^. Currently, the COVID-19 vaccine with simian adenovirus^41,42^ has been approved for clinical use. This successful development of vaccines with simian adenovirus and our previous finding that Mm32 was able to use several mammalian-derived coxsackie virus and adenovirus receptors (CXADRs) as entry factors^21^ suggest that BtAdV possesses potential as a vector platform for recombinant OVs or vaccines for dogs, humans, and other animals.

BAC-based reverse genetics systems for adenoviruses have been reported in human adenovirus^43^, simian adenovirus^44^, and canine adenovirus^45^. BAC clones with viral genome sequences are maintained as single or double copies, providing stability to the *Escherichia coli* host. Furthermore, using BAC recombination methods, regions of interest from the viral genome in the BAC clone can be easily manipulated. Although the detection and isolation of BtAdVs have been reported so far^17–21^, to our knowledge, this is the first report of a BAC-based reverse genetics system for BtAdVs, and provides a powerful tool for basic studies on BtAdVs, as well as for the construction of a gene transfer vector or an oncolytic virus. Advanced molecular studies on viruses of bat origin are expected to further analyze their zoonotic potential.

Modification or replacement of E1Ap is one of the most promising methods for constructing oncolytic adenoviruses. Most oncolytic HAdVs, whose E1Ap was replaced with a tumor-specific promoter, were highly attenuated in normal cells, but often replicated less efficiently than the wild-type virus in tumor cells^9^. This decrease in tumor cells limits the therapeutic efficacy of OVs in clinical settings. Our generated BtAdV Mm32-E1Ap + cTERTp grew to higher titers than Mm32-rWT in canine tumor cells, whereas it grew to nearly the same titer as Mm32-rWT in normal canine cells. These findings suggest that the anti-tumor effect of Mm32-E1Ap + cTERTp is higher than that of Mm32-rWT. The activity of the chimeric promoter in Mm32-E1Ap + cTERTp was higher than that of E1Ap in most tumor cells, indicating a possible synergistic effect between E1Ap and cTERTp, which might have enhanced the growth of Mm32-E1Ap + cTERTp in canine tumor cells. Previous reports have shown that an enhancer and several transcription initiation sites in the left end of the adenoviral genome may impair the fidelity of the inserted tumor-specific promoter^46^. To stop the transcription initiation and to block the enhancer effect, inserting a poly A signal and an insulator upstream of the cTERTp in the Mm32-E1Ap + cTERTp^47^ or deleting more regions of E1Ap while retaining the genome packaging signal would be a promising strategy. This would improve the fidelity of cTERTp and the tumor-specificity of Mm32-E1Ap + cTERTp. Also, deleting pRB-binding region of E1A protein or E1B-55 K homolog, reported in human adenovirus-based oncolytic virus^7,8^, might lead to enhanced tumor-specificity and concomitantly decrease its growth in normal cells.

Notably, a previous report hypothesized that interspecies transmission of BtAdV to carnivores had occurred in the past and that CAdV might have originated from BtAdV, as BtAdV was phylogenetically close to CAdV^48^. Our finding that BtAdVs could replicate in various canine cell cultures, including normal cells, may support this hypothesis. The pathogenicity of BtAdVs in bats and other animals, including dogs, remains unknown. Experimental infections with our BtAdV strain and its recombinant virus to evaluate their pathogenicity in dogs will be required to assess the utility of Mm32-E1Ap + cTERTp as an OV candidate for canine cancers. Previous studies using a reovirus-based OV strategy showed that reovirus serotype 3, a promising OV for human tumors as well as for canine tumors, replicated well in canine bone marrow-derived mast cells in vitro (10^8–9^ TCID_50_/mL)^49^, but did not cause any disease or severe adverse effects in a canine clinical study^50^. This report suggests that in vitro studies do not necessarily reflect in vivo effects, emphasizing the importance and necessity of evaluating OV candidates in vivo. In particular, to evaluate the safety of BtAdV-based OV, we need to test it in dogs by directly injecting OV candidates into tumors, since using nude mice xenograft models would not be feasible to test OV candidates, as they do not cause infectivity in mice.

In conclusion, we generated a BtAdV-based OV candidate targeting canine tumors. Further preclinical assessments, including in vivo studies, may lead to the production of safe and effective BtAdV-Mm32-based OV candidates, followed by clinical studies for alternative canine cancer therapy in dogs.

## Methods

### Cells and viruses

MDCK cells were obtained from the American Type Culture Collection (CCL-34). MDCK cells and MDCK-Mm32-E1-28 cells (described below) were maintained in Dulbecco’s modified Eagle medium (DMEM) supplemented with 5% fetal bovine serum (FBS), penicillin (100 U/mL), and streptomycin (0.1 mg/mL) at 37 °C with 5% CO_2_. Five canine mammary tumor cell lines (CTBm^23^, CTBp^23^, CIPm^23^, CIPp^23^, and CHMm^23^), five canine melanoma cell lines (CMeC1^24^, CMeC2^24^, KMeC^24^, LMeC^24^, and CMM1^25^), three canine osteosarcoma cell lines (HMPOS^26^, HOS^27^, and OOS^27^), and a canine mast cell tumor cell line (LuMC^28^) were maintained in RPMI 1640 medium supplemented with 10% FBS, penicillin, and streptomycin at 37 °C with 5% CO_2_. Two lots of primary canine kidney fibroblast cells (DK-P lot1 and DK-P lot2), which had been prepared and stored in our laboratory, were used as normal canine cells and were maintained in DMEM supplemented with 10% FBS, penicillin, and streptomycin at 37 °C in 5% CO_2_. CAdV1 [strain D43 (LC557010)], CAdV2 [strain A2 (LC557011)], and BtAdVs [strain Vs9 (LC385827) and Mm32 (LC385828)], which we had isolated from Japanese microbats in our previous study^21^, were propagated in MDCK cells in DMEM supplemented with 1% FBS. We did not handle any microbats in the current study. Mm32-ΔE1-Venus and Mm32-E1Ap + cTERTp (described below) were propagated in the MDCK-Mm32-E1-28 cells.

### Cloning of the BtAdV genome into a BAC vector

This method essentially followed our own previous report on canine adenoviruses^45^. The primers used in this study are listed in Supplementary Table S1. To add 50-bp homologous arms and restriction enzyme sites to the galactokinase (galK) expression cassette, the cassette was amplified from pGalK (National Cancer Institute) using the primers Vs9-ITR-GalK F1 and Vs9-ITR-GalK R1 or Mm32-ITR-GalK F1 and Mm32-ITR-GalK R1. The PCR products were then amplified using the primers Vs9-ITR FR2 or Mm32-ITR FR2. The resultant fragments were digested with BamHI and cloned into BamHI-digested pSMART BAC using the CopyRight v2.0 BAC Cloning Kit (Lucigen). The plasmids were then used to transform BAC-Optimized Replicator v2.0 Electrocompetent cells (Lucigen) using a MicroPulser (BioRad) at preset condition Ec1. The resultant BAC vectors were named pBAC-Vs9-ITR-GalK and pBAC-Mm32-ITR-GalK.

To clone the full-length Vs9 and Mm32 genomes, we used *Escherichia coli* strain SW102 (National Cancer Institute), which contains a fully functional galactose operon (except with galK deleted), and expressed red recombinase at 42 °C^51^. Briefly, SW102 cells containing pBAC-Vs9-ITR-GalK or pBAC-Mm32-ITR-GalK were incubated overnight in 1 mL of Luria–Bertani (LB) medium supplemented with 12.5 µg/mL of chloramphenicol (CP) at 32 °C with shaking at 180 rpm. The following day, the overnight culture (0.5 mL) was diluted in 10 mL of CP-containing LB medium and incubated at 32 °C for 1.5–2 h until the OD_600_ reached 0.4–0.6. Red recombinase was induced by heat shock at 42 °C for 15 min. Thereafter, the cells were chilled on ice for 10–15 min and washed three times with cold H_2_O. The cell pellet was resuspended in 30–50 µL of cold H_2_O and kept on ice. A 25-µL aliquot of the cells was mixed with 300 ng of Vs9 DNA or Mm32 DNA, and the mixture was electroporated using a MicroPulser (BioRad) at preset condition Ec1. After electroporation, the cells were recovered in CP-containing LB medium at 32 °C for 4.5 h. The cells were then washed twice with M9 medium and plated onto M63 minimum medium plates supplemented with glycerol, leucine, biotin, 2-deoxygalactose, and CP. The plates were incubated at 32 °C for 3 days, after which the bacterial colonies were screened by PCR using the following primers: SL1 and Vs9 408 R, SL1 and Vs9 30630 F, pSMART BAC 159 R and Vs9 408 R, and pSMART BAC 159 R and Vs9 30630 F for Vs9, and SL1 and Mm32 456 R, SL1 and Mm32 31375 F, pSMART BAC 159 R and Mm32 456 R, and pSMART BAC 159 R and Mm32 31,375 F for Mm32. The positive BACs were then digested with restriction enzymes, and their sizes were confirmed by electrophoresis. The resultant BACs were named pBAC-Vs9 and pBAC-Mm32.

### Generation of recombinant BtAdVs

This method essentially followed our own previous report on canine adenoviruses^45^. To generate recombinant BtAdVs, the respective BACs were digested with PvuI. Thereafter, the linearized viral genomic DNA was transfected into MDCK cells using polyethylenimine (Polysciences) according to the manufacturer’s instructions. At 5–6 days post-transfection, the medium and cells were frozen and thawed three times, and the supernatants obtained from centrifugation of cell cultures were collected and used to infect MDCK cells. Once 50%–80% of the cells showed cytopathic effects, the culture supernatants were collected and stored at − 80 °C. Genomic DNA was extracted from recombinant wild-type (rWT) BtAdVs, and the restriction patterns were compared with those of the genomic DNA from wild-type (WT) BtAdVs.

### Modification of the Mm32 genome in the BAC clone

This method essentially followed our own previous report on canine adenoviruses^45^. To construct the Mm32 infectious clone with the E1 region deleted, galK-Kn cassettes were amplified from pGalK-Kn^45^, which encodes the GalK and kanamycin resistance genes under the same promoter, using the primers Mm32-E1-GalK-Kn F and Mm32-E1-GalK-Kn R. SW102 cells containing pBAC-Mm32 were heat shocked to induce red recombinase, followed by electroporation with 100 ng of the PCR product. The recovered cells were plated on LB medium plates containing CP and 25 µg/mL kanamycin. After 18–24 h, the bacterial colonies were passaged on MacConkey medium plates supplemented with CP and d-galactose. Then, the red bacterial colonies that had formed on the plates, which were GalK-positive SW102 cells, were transferred to LB medium containing CP and kanamycin and incubated overnight. The following day, the overnight culture (0.5 mL) was diluted in 10 mL of CP-containing LB medium and incubated at 32 °C for 1.5–2 h. Then, red recombinase was induced by heat shock. The Venus expression cassette was amplified from the Venus expression plasmid (pcDNA3.1-Venus) using the primers Venus cassette F and Venus cassette R. The purified PCR product (100 ng) was then introduced into the cells to replace the galK-Kn cassettes. After incubation for 4.5 h, the cells were washed twice with M9 medium and plated onto M63 minimum medium plates supplemented with glycerol, leucine, biotin, 2-deoxygalactose, and CP. Bacterial colonies were screened and a single positive colony was selected. The resultant BAC was named pBAC-Mm32-ΔE1-Venus.

To construct the Mm32 infectious clone with cTERTp inserted into the E1A regulatory region, the galk-Kn expression cassette was amplified using the primers Mm32-E1-GalK-Kn F and Mm32-E1Ap-GalK-Kn R. Galk-Kn expression cassette was inserted into pBAC-Mm32. Then, cTERTp was amplified from pGL4.10-cTERTp (described below) using the primers Mm32-E1Ap + cTERTp F and Mm32-E1Ap + cTERTp R, and the galk-Kn cassette was replaced with cTERTp. The resultant BAC was named pBAC-Mm32-E1Ap + cTERTp.

### Propagation of E1-modified Mm32 mutant

This method essentially followed our own previous report on canine adenoviruses^45^. To construct the E1 expression plasmid, the E1 region of Mm32 was amplified by PCR and then cloned into HindIII-XhoI-digested pKS336 (AF403737) to generate pKS-Mm32-E1. To detect the E1A protein, a 3xFLAG tag was fused to the N-terminus of the E1A protein. The resultant plasmid was linearized through BsaI digestion and introduced into MDCK cells using polyethylenimine. At 24 h post-transfection, 15 µg/mL blasticidin was added. After 4–5 days of incubation, the cells were subcultured for colony formation. These clones were screened, and a single clone, named MDCK-Mm32-E1-28, was chosen for use in this study. E1A expression was confirmed by indirect immunofluorescence assay (IFA) using a mouse anti-DDDDK-tag monoclonal antibody (MBL) and a goat polyclonal anti-mouse IgG antibody conjugated with a fluorophore (Alexa Fluor 488). The expression of canine glyceraldehyde 3-phosphate dehydrogenase (cGAPDH) and E1B was confirmed by reverse transcription (RT)-PCR using the primers cGAPDH F and cGAPDH R and Mm32-E1B F and Mm32-E1B R, respectively.

To generate recombinant Mm32, pBAC-Mm32-ΔE1-Venus, and pBAC-Mm32-E1Ap + cTERTp was first digested with PvuI, followed by transfection of the linearized viral genomic DNAs into MDCK-Mm32-E1-28 cells using polyethylenimine. At 5–6 days post-transfection, the medium and cells were frozen and thawed three times, and the supernatants obtained from centrifugation of the cell cultures were collected and used to infect the MDCK-Mm32-E1-28 cells. Once 50%–80% of the cells showed cytopathic effects, the culture supernatants were collected and stored at − 80 °C.

### Growth of recombinant viruses in the cell culture

This method essentially followed our own previous report on canine adenoviruses^45^. MDCK cells were infected with BtAdV-WT or BtAdV-rWT viruses at a multiplicity of infection (MOI) of 0.01. After incubation at 37 °C for 1 h, the inocula were completely removed. The cells were washed twice using DMEM supplemented with 1% FBS and maintained in DMEM supplemented with 1% FBS, and the culture supernatants were collected daily for up to 5 days post-infection (dpi). To determine the growth properties of CAdVs and BtAdVs in canine tumor cells, canine tumor cells were infected with CAdV1, CAdV2, Vs9, or Mm32 at an MOI of 5. After incubation at 37 °C for 1 h, the inocula were completely removed. The cells were then washed twice with DMEM supplemented with 1% FBS, and all cells were maintained in RPMI 1640 medium supplemented with 10% FBS, except for CHMm cells, which were maintained in DMEM supplemented with 10% FBS. At 3 dpi, the medium and cells were frozen and thawed three times, and the supernatants obtained from centrifugation of the cell cultures were collected. To measure viral titers, plaque assays were performed using MDCK cells. After virus adsorption to the cells in 12-well plates at 37 °C for 1 h, the inocula were removed, and the cells were overlaid with Eagle’s minimal essential medium supplemented with 1% FBS and 0.8% agarose. At 4 dpi, the agarose was removed, the plaques were fixed with methanol, and stained with 0.1% crystal violet to count the plaque numbers (Supplementary Fig. S5).

To determine the growth properties of Mm32-rWT and Mm32-E1Ap + cTERTp in the canine tumors and normal cells, CTBm, CIPm, CMeC1, LMeC, HMPOS, HOS, DK-P lot1, and DK-P lot2 cells were infected with Mm32-rWT or Mm32-E1Ap + cTERTp at an MOI of 0.1. All cells were maintained in RPMI 1640 medium supplemented with 10% FBS, except for DK-P lot1 and DK-P lot2 cells, which were maintained in DMEM supplemented with 10% FBS. At 3 dpi, the medium and cells were frozen and thawed three times, and the supernatants obtained by centrifuging the cell cultures were collected. To measure viral titers, plaque assays were performed using MDCK-Mm32-E1-28 cells (Supplementary Fig. S5).

To determine the growth properties of Mm32-ΔE1-Venus, MDCK and MDCK-Mm32-E1-28 cells were infected with Mm32-rWT or Mm32-ΔE1-Venus at an MOI of 0.2. After incubation at 37 °C for 1 h, the inocula were completely removed. The cells were then washed twice with DMEM supplemented with 1% FBS, and the viral titers (median tissue culture infectious dose [TCID_50_/mL]) were measured using MDCK-Mm32-E1-28 cells.

### Cytotoxicity assay

Cells in 24-well plates were infected with CAdV1, CAdV2, Vs9, Mm32, Mm32-rWT, or Mm32-E1Ap + cTERTp at various MOIs. At 5 dpi, cells, except for LuMC cells, were fixed with methanol and stained with 0.1% crystal violet. Excess crystal violet was removed by washing with water. The plates were dried and scanned using a GT-X820 scanner (EPSON). LuMC cells were stained with 0.5% trypan blue, and the viable cells were counted.

### Luciferase reporter assay

The cTERTp, the E1A regulatory region of Mm32, and the fused promoter (cTERTp was inserted into the E1A regulatory region) were cloned into pGL4.10 [luc2] (AY738222), which encodes firefly luciferase, and the resultant plasmids were named pGL4.10-cTERTp, pGL4.10-E1Ap, and pGL4.10-E1Ap + cTERTp. The pRL-TK plasmid (AF025846) encoding Renilla luciferase under the HSV-TK promoter was used as an internal control to normalize transfection efficiency. pGL4.10-cTERTp, pGL4.10-E1Ap, pGL4.10-E1Ap + cTERTp, and pGL4.10 [luc2], together with pRL-TK, were transfected into cells using polyethylenimine for CTBm, CIPm, and LMeC cells; The plamids were transfected into DK-P lot1, DK-P lot2, CMeC1, HMPOS, and HOS cells using the TransIT-X2 system (Mirus Bio LLC) according to the manufacturer’s instructions. At 24 h post-transfection, the luciferase activity of the cell lysates was measured and standardized to the activity of Renilla luciferase using the Dual-Glo Luciferase Assay System (Promega) on an ARVO X2 microplate luminometer (PerkinElmer Japan). All experiments were performed in triplicate.

### Statistical analysis

Datasets on the viral growth kinetics in MDCK and MDCK-Mm32-E1-28 cells were analyzed using mixed ANOVA (a linear mixed model) (Figs. 2 and 3). Paired data on the viral growth at each time-point in these cells were analyzed using the Holm method as a post-hoc test (Fig. 3) and those of Mm32-rWT and Mm32-E1Ap + cTERTp in canine tumor cells or normal cells were analyzed using Student’s t-test (Fig. 5A). Datasets on the viral growth of CAdVs and BtAdVs in canine tumor cells (Fig. 1A) and from the luciferase reporter assay (Fig. 4B) were analyzed using the Holm method, with two-tailed analysis, to determine the statistical significance of the differences.

## Supplementary Information

Supplementary Information.
