# Supplementary material for: A potential bat adenovirus-based oncolytic virus targeting canine cancers
Source: Sci Rep. 2021 Aug 18;11:16706. doi: 10.1038/s41598-021-96101-4 (PMC8373906; doi:10.1038/s41598-021-96101-4)
Supplement: Supplementary file 1 — Supplementary Information. [file 41598_2021_96101_MOESM1_ESM.pdf]

Supplementary Information

A potential bat adenovirus-based oncolytic virus targeting canine cancers

Hiromichi Matsugo, Tomoya Kobayashi-Kitamura, Haruhiko Kamiki, Hiroho Ishida, Wataru Sekine, Akiko Takenaka-Uema, Takayuki Nakagawa, Shin Murakami & Taisuke Horimoto

Table S1. Primers used in this study

Primer	Sequence (5′ – 3′)	Sequence capitalized
Vs9-ITR-GalK F1	GGACAAAGAGGTGTGGCCTAATTTGGTGGGCGcctgttgacaattaatcatcg	inverted terminal repeats (ITR) sequence
Vs9-ITR-GalK R1	GGACAAAGAGGTGTGGCCTAATTTGGTGGGCGtcagcactgtcctgctccttg	inverted terminal repeats (ITR) sequence
Mm32--ITR-GalK F1	GTGCAAAAAGGTGTGGCCTAATTTTGAGGGCGcctgttgacaattaatcatcg	inverted terminal repeats (ITR) sequence
Mm32-ITR-GalK R1	GTGCAAAAAGGTGTGGCCTAATTTTGAGGGCGtcagcactgtcctgctccttg	inverted terminal repeats (ITR) sequence
Vs9-ITR FR2	atctGGATCCTTAATTAACGATCGcatcatcaataatacacaggacaaagaggtgtggcctaatttggtg	restriction enzyme sites
Mm32-ITR FR2	atctGGATCCTTAATTAACGATCGcatcatcaataatatacagtgcaaaaaggtgtggcctaattttgag	restriction enzyme sites
SL1	cagtccagttacgctggagtc	
SMART BAC 159R	aacctcttacgtgccgatcag	
Vs9 408R	cgcactcaagagtggcactc	
Vs9 30630F	gtctcacagactgtgctctgg	
Mm32 456R	agcacgtatttaaagtgcac	
Mm32 31375F	ggttcctcaaaagcggtgcac	
Mm32-E1-GalK-Kn F	GCTCCTTCTGGGCAGAGTCGTAGTTAATCATTATTTTTCCGGGAGTGCACcctgttgacaattaatcatcg	homologous sequence
Mm32-E1-GalK-Kn R	AGTCCAAACAGGCAGTTCCGGGGATGAAACACCCCCCGCCTCTTTCCCAGtcagaagaactcgtcaagaagg	homologous sequence
Venus cassette F	AGTCCAAACAGGCAGTTCCGGGGATGAAACACCCCCCGCCTCTTTCCCAGgacattgattattgactag	homologous sequence
Venus cassette R	GCTCCTTCTGGGCAGAGTCGTAGTTAATCATTATTTTTCCGGGAGTGCACccatagagcccaccgcatcc	homologous sequence
Mm32-E1Ap-GalK-Kn R	TCGCTCCGCACTCAAGAGTAGTCCTCTTTCGGTTTGAGCACGTATTTAAAtcagaagaactcgtcaagaagg	homologous sequence
Mm32-cTERTp F	GCTCCTTCTGGGCAGAGTCGTAGTTAATCATTATTTTTCCGGGAGTGCACctgtgacagggacaaacccaag	homologous sequence
Mm32-cTERTp R	GCTCCGCACTCAAGAGTAGTCCTCTTTCGGTTTGAGCACGTATTTAAAggccggggaggggcggggtccgcgg gaggaag	homologous sequence
cGAPDH F	cgatggtgaaggtcggagtg	
cGAPDH R	actggaacatgtacaccatgtagtt	
Mm32-E1B F	tagttagacgccaggaccag	
Mm32-E1B R	ggtgagaacacacatgcacg	

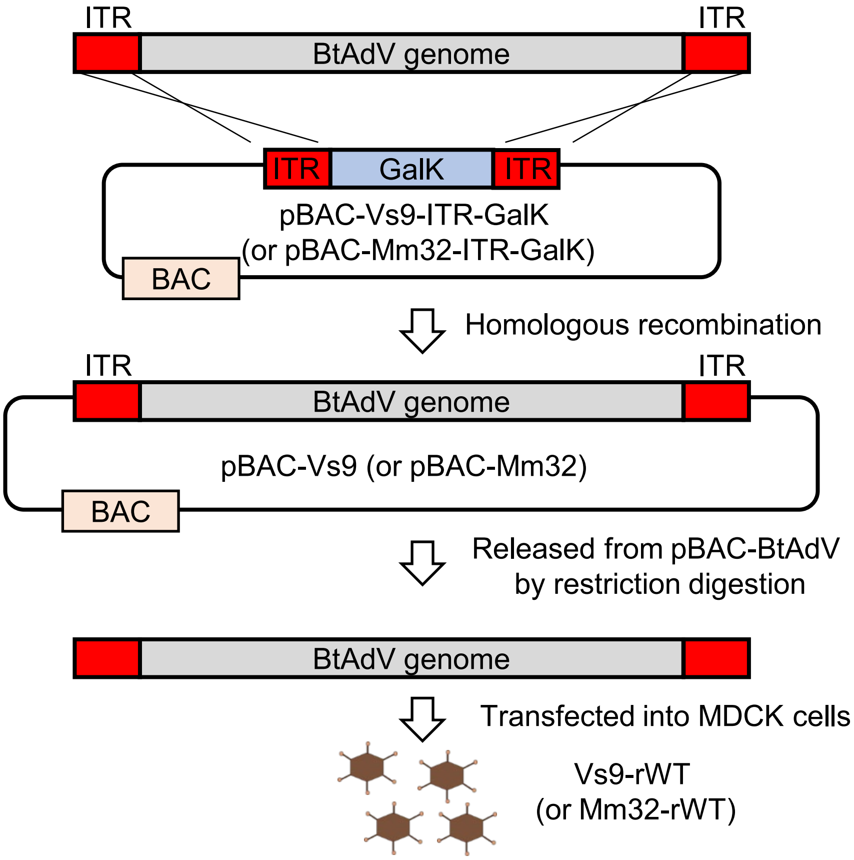

Fig. S1. Cloning of the BtAdV genome into BAC vectors. Vs9 (or Mm32) DNA was introduced into SW102 cells harboring pBAC-Vs9-ITR-GalK (or pBAC-Mm32-ITR-GalK). The plasmid contains a galactokinase (galK) expression cassette flanked by approximately 50-bp of inverted terminal repeats (ITR) of the viral genome. After homologous recombination by Red recombinase, cells were selected using GalK, and pBAC-Vs9 (or pBAC-Mm32) was obtained. Then, this BAC clone was digested using a restriction enzyme and transfected into MDCK cells, following which the recombinant wild-type virus, Vs9-rWT (or Mm32-rWT), was rescued.

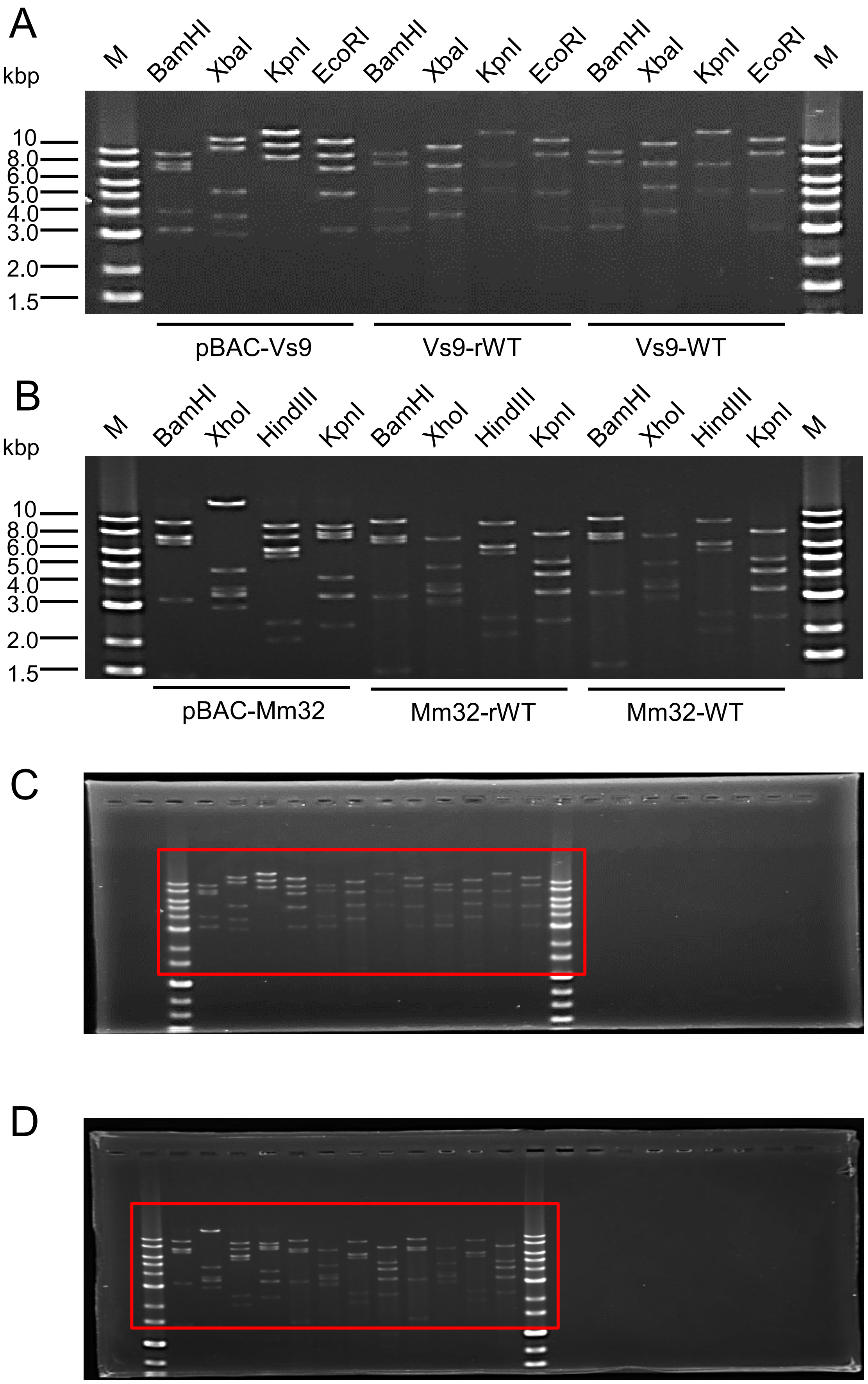

A
M BamHI XbaI KpnI EcoRI BamHI XbaI KpnI EcoRI BamHI XbaI KpnI EcoRI M
kbp
10
8.0
6.0
5.0
4.0
3.0
2.0
1.5
pBAC-Vs9
Vs9-rWT
Vs9-WT
B
M BamHI XhoI HindIII KpnI BamHI XhoI HindIII KpnI BamHI XhoI HindIII KpnI M
kbp
10
8.0
6.0
5.0
4.0
3.0
2.0
1.5
pBAC-Mm32
Mm32-rWT
Mm32-WT
C
D

Fig. S2. Restriction digestion of the genomes of wild-type (WT), recombinant wild-type (rWT) viruses, and BAC clone. (A) pBAC-Vs9, Vs9-rWT, and Vs9-WT DNAs were digested using BamHI, XbaI, KpnI, and EcoRI, respectively. The predicted molecular sizes of the digested fragments of pBAC-Vs9 are 9764, 8248, 7620, 4046, 3215, 3171, 1153, 813, 674, and 168 bp for BamHI; 13775, 11062, 5385, 3796, 3019, 826, 775, and 234 bp for XbaI; 17910, 11709, and 9253 bp for KpnI; and 12780, 9460, 7592, 5161, 3142, 559, and 178 bp for EcoRI. The predicted molecular sizes of the digested fragments of the viral genomes are 9764, 8248, 4046, 3200, 3171, 1153, 794, 674, and 168 bp for BamHI; 11062, 7848, 5395, 3796, 1292, 826, 765, and 234 bp for XbaI; 17910, 7967, and 5341 bp for KpnI; and 12780, 9460, 5161, 3142, 530, and 145 bp for EcoRI. M: 1-kb DNA ladder marker. (B) pBAC-Mm32, Mm32-rWT, and Mm32-WT DNA were digested using BamHI, XhoI, HindIII, and KpnI, respectively. The predicted molecular sizes of the digested fragments of pBAC-Mm32 are 9660, 7620, 7350, 6944, 3260, 1445, 1427, 758, 530, 380, and 30 bp for BamHI; 17886, 4757, 3733, 3503, 3443, 2960, 1388, 809, 751, and 174 bp for XhoI; 9066, 7606, 6289, 6213, 5742, 2454, and 2034 bp for HindIII; and 8880, 7960, 7462, 4212, 3334, 3299, 2342, 1063, 840, and 12 bp for KpnI. The predicted molecular sizes of the digested fragments of the viral genomes are 9660, 7350, 6929, 3260, 1427, 1426, 758, 530, 380, and 30 bp for BamHI; 7132, 4757, 3733, 3503, 3443, 3100, 2960, 1388, 809, 751, and 174 bp for XhoI; 9066, 6289, 6191, 5716, 2454, and 2034 bp for HindIII; and 7462, 4972, 4214, 4212, 3334, 3299, 2342, 1063, 840, and 12 bp for KpnI. M: 1-kb DNA ladder marker. The predicted restriction digestion sizes of the plasmid backbone are; no site (supercoiled ~7620bp) with XhoI, 7620bp by one site with BamHI, KpnI, or XbaI, 7592bp and 28bp by two sites with EcoRI, or 7606bp and 14bp by two sites with HindIII, indicating that they would not affect any digestion profiles of pBAC-Mm32 and pBAC-Vs9.The figure image was made directly from one gel each (C and D, used for A and B, respectively).

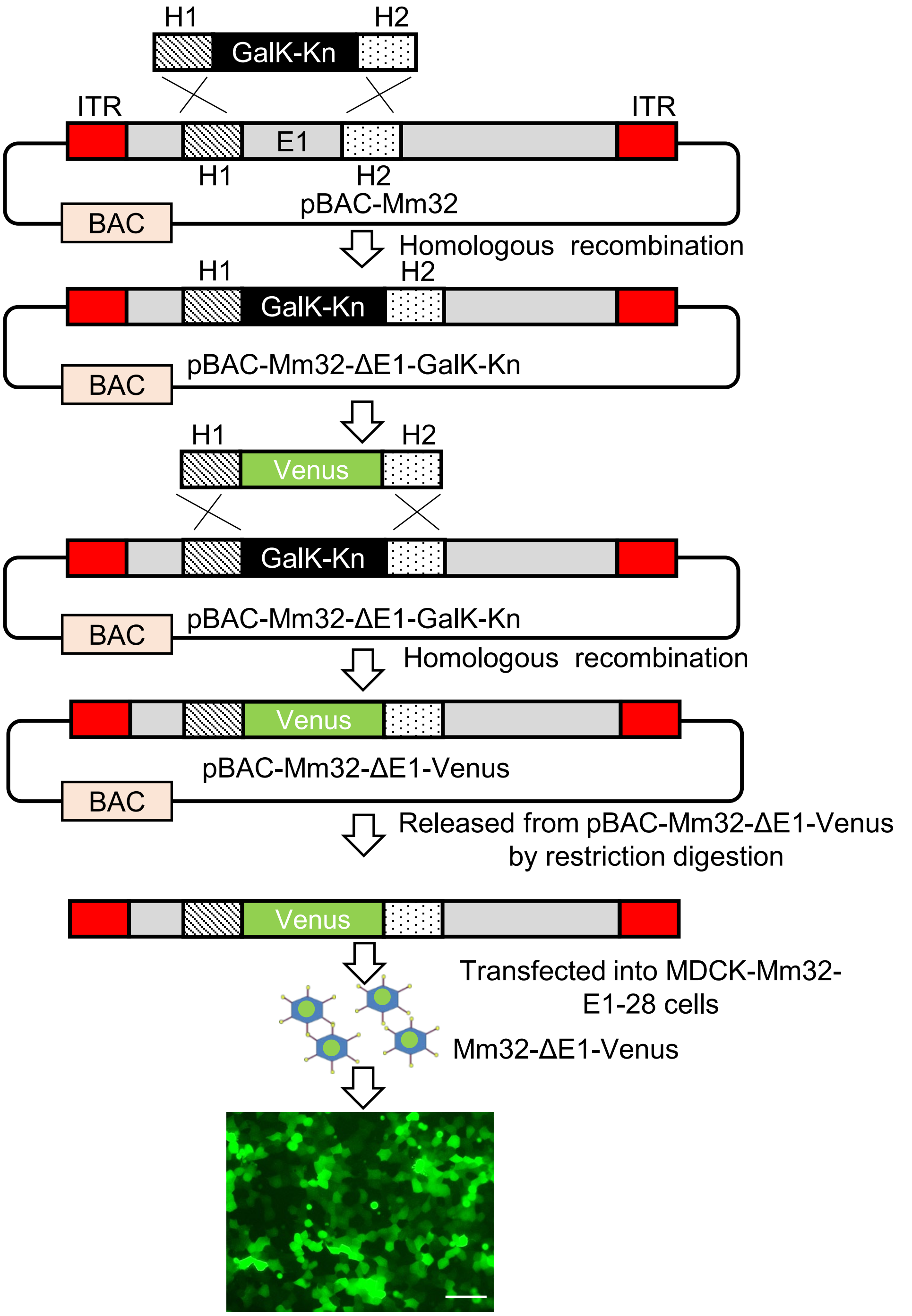

Fig. S3. Generation of the E1-deleted Mm32 mutant. The E1 region of pBAC-Mm32 was replaced with the Venus expression cassette. The galK and kanamycin resistance gene (galK-Kn) expression cassette, flanked by homologous region 1 (H1) and region 2 (H2), was generated by PCR and introduced into SW102 cells harboring pBAC-Mm32. After homologous recombination, cells were selected by GalK and Kn, to obtain cells harboring pBAC-Mm32-ΔE1-GalK-Kn. The Venus expression cassette flanked by H1 and H2 was generated by PCR and introduced into SW102 cells harboring pBAC-Mm32-ΔE1-GalK-Kn. After homologous recombination, cells were selected using GalK to obtain cells harboring pBAC-Mm32-ΔE1-Venus. Mm32-ΔE1-Venus was rescued through the transfection of restriction enzyme-digested pBAC-Mm32-ΔE1-Venus into MDCK-Mm32-E1-28 cells. After the MDCK cells had been infected with Mm32-ΔE1-Venus at an MOI of 4, Venus expression was observed by fluorescence microscopy at 18 hours post-infection. Scale bar indicates 100 μm.

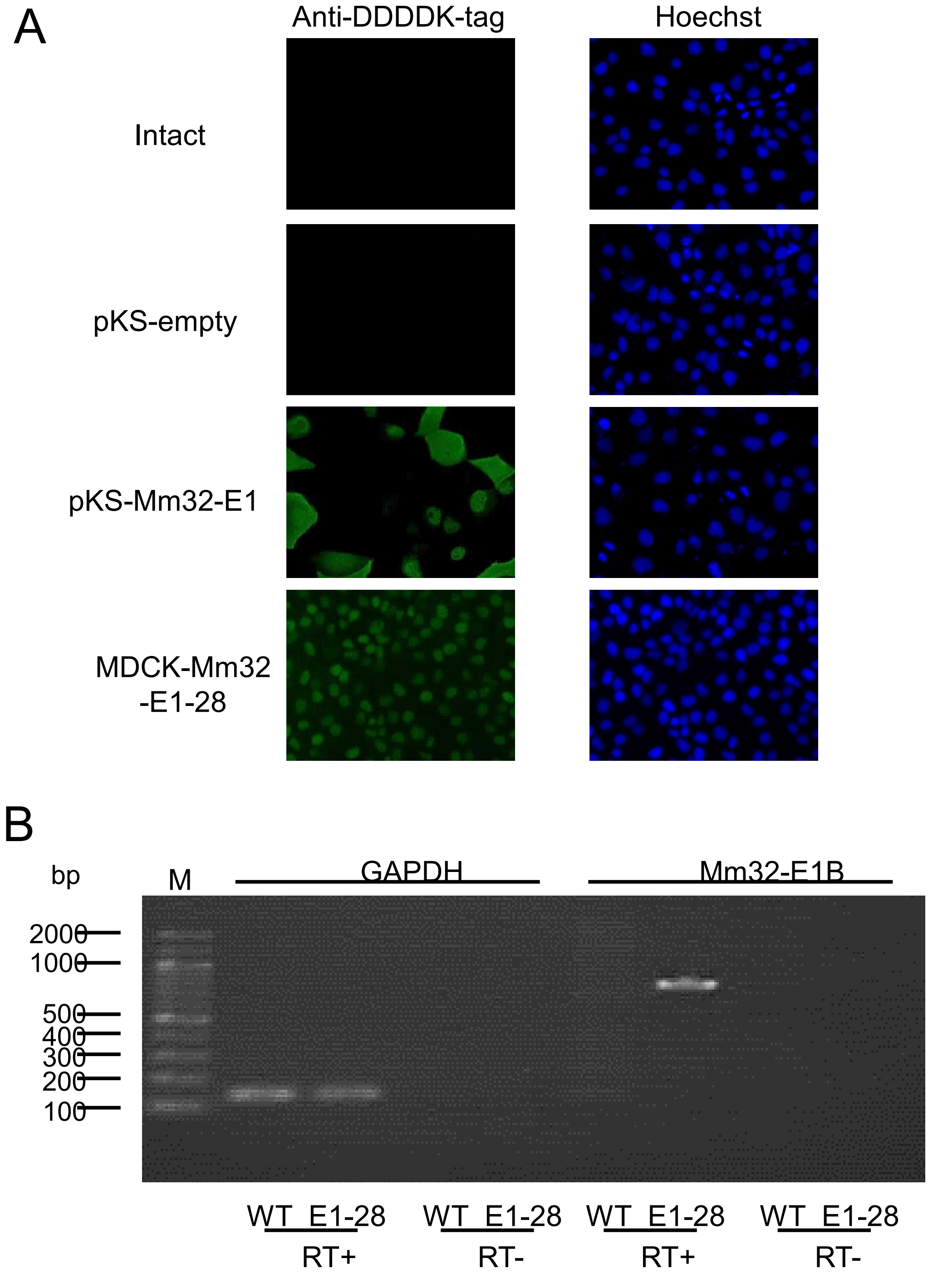

Fig. S4. Mm32 E1 gene expression in MDCK-Mm32-E1-28 cells. (A) Detection of Mm32 E1A protein using indirect immunofluorescence assay. Mm32 E1A protein in MDCK-Mm32-E1A-28 cells and pKS-Mm32-E1-transfected MDCK cells were detected at 24 hours post-transfection using a mouse monoclonal anti-DDDDK-tag antibody and a goat polyclonal anti-mouse IgG antibody conjugated with a fluorophore (Alexa 488). Intact MDCK cells and pKS-empty-transfected cells were used as negative controls. The nuclei were stained with Hoechst. (B) Detection of Mm32 E1B mRNA using RT-PCR. RNA was extracted from wild-type (WT) MDCK or MDCK-Mm32-E1-28 cells (E1-28). RT-PCR was performed using primers specific for canine glyceraldehyde 3-phosphate dehydrogenase (GAPDH), and Mm32-E1B, with (+) or without (-) reverse transcriptase (RT). M: 100-bp DNA ladder marker.

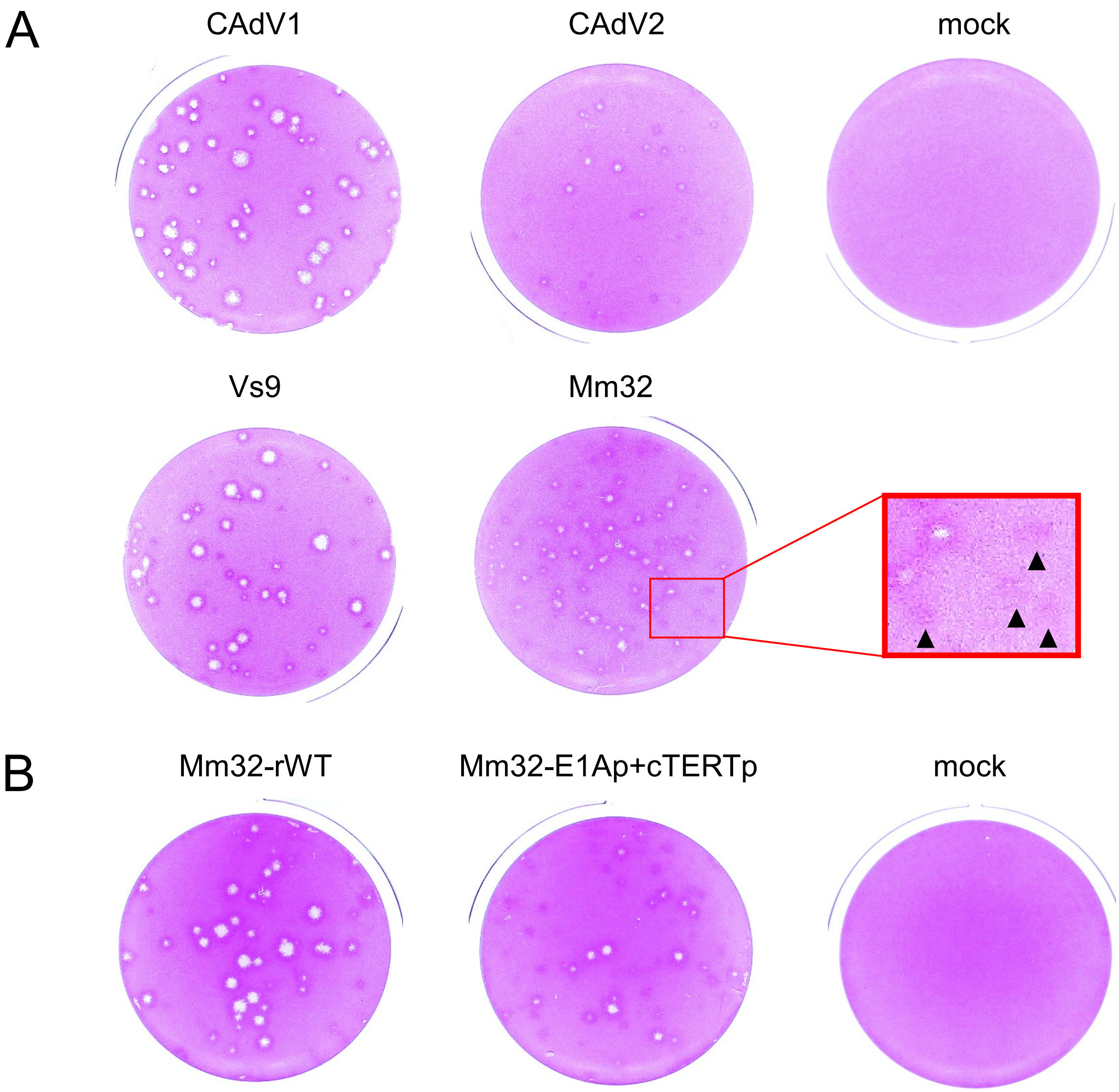

Fig. S5. Plaque morphorogy of each virus. After virus adsorption to the (A) MDCK or (B) MDCK-Mm32-E1-28 cells in 12-well plates at 37°C for 1 h, the inocula were removed, and the cells were overlaid with Eagle's minimal essential medium supplemented with 1% FBS and 0.8% agarose. At 4 dpi, the agarose was removed, the plaques were fixed with methanol, and stained with 0.1% crystal violet. Mock-infected cells (mock) are also shown. Arrow heads indicate examples of the small plaques, which were included in titer quantification, in an enlarged image (red square).
